# RNAseq and targeted metabolomics implicate RIC8 in regulation of energy homeostasis, amino acid compartmentation, and asexual development in *Neurospora crassa*

**DOI:** 10.1128/mbio.03133-24

**Published:** 2024-11-18

**Authors:** Monique Quinn, Alexander J. Carrillo, Lida Halilovic, Katherine A. Borkovich

**Affiliations:** 1 Department of Microbiology and Plant Pathology, University of California, Riverside, California, USA; University of California, Berkeley, Berkeley, California, USA

**Keywords:** heterotrimeric G proteins, nonreceptor guanine nucleotide exchange factor, filamentous fungi, asexual development, amino acid compartmentation, arginine

## Abstract

Heterotrimeric G protein signaling pathways control growth and development in eukaryotes. In the multicellular fungus *Neurospora crassa*, the guanine nucleotide exchange factor RIC8 regulates heterotrimeric Gα subunits. In this study, we used RNAseq and liquid chromatography-mass spectrometry (LC-MS) to profile the transcriptomes and metabolomes of *N. crassa* wild type, the Gα subunit mutants Δ*gna-1* and Δ*gna-3*, and Δ*ric8* strains. These strains exhibit defects in growth and asexual development (conidiation), with wild-type and Δ*gna-1* mutants producing hyphae in submerged cultures, while Δ*gna-3* and Δ*ric8* mutants develop conidiophores, particularly in the Δ*ric8* mutant. RNAseq analysis showed that the Δ*gna-1* mutant possesses 159 mis-regulated genes, while Δ*gna-3* and Δ*ric8* strains have more than 1,000 each. Many of the mis-regulated genes are involved in energy homeostasis, conidiation, or metabolism. LC-MS revealed changes in levels of primary metabolites in the mutants, with several arginine metabolic intermediates impacted in Δ*ric8* strains. The differences in metabolite levels could not be fully explained by the expression or activity of pathway enzymes. However, transcript levels for two predicted vacuolar arginine transporters were affected in Δ*ric8* mutants. Analysis of arginine and ornithine levels in transporter mutants yielded support for altered compartmentation of arginine and ornithine between the cytosol and vacuole in Δ*ric8* strains. Furthermore, we validated previous reports that arginine and ornithine levels are low in wild-type conidia. Our results suggest that RIC8 regulates asexual sporulation in *N. crassa* at least in part through altered expression of vacuolar transporter genes and the resultant mis-compartmentation of arginine and ornithine.

**IMPORTANCE:**

Resistance to inhibitors of cholinesterase-8 (RIC8) is an important regulator of heterotrimeric Gα proteins in eukaryotes. In the filamentous fungus *Neurospora crassa*, mutants lacking ric8 undergo inappropriate asexual development (macroconidiation) during submerged growth. Our work identifies a role for RIC8 in regulating expression of transporter genes that retain arginine and ornithine in the vacuole (equivalent of the animal lysosome) and relates this function to the developmental defect. Arginine is a critical cellular metabolite, both as an amino acid for protein synthesis and as a precursor for an array of compounds, including proline, ornithine, citrulline, polyamines, creatine phosphate, and nitric oxide. These results have broad relevance to human physiology and disease, as arginine modulates immune, vascular, hormonal, and other functions in humans.

## INTRODUCTION

All organisms depend on cell surface receptors to sense their external environment and modulate growth and development (1). In eukaryotes, specialized plasma membrane proteins called G protein-coupled receptors (GPCRs) recognize external stimuli (2). GPCRs transduce an external signal (ligand) to intracellular signaling pathways via heterotrimeric G proteins (2). The heterotrimeric G protein is made up of three subunits: α, β, and γ. The Gα subunit can be bound to GDP or GTP. When bound to GDP, the Gα is inactive and is in a complex with the Gβγ dimer and a GPCR (2, 3). When bound to GTP, the Gα disassociates from the Gβγ dimer and both can regulate downstream signaling (1, 3, 4). Non-receptor GEFs, such as resistance to inhibitors of cholinesterase 8 (RIC8), also facilitate GDP/GTP exchange on Gα subunits and their return to the activated state (5, 6).

In the multicellular fungus *Neurospora crassa*, heterotrimeric G protein signaling regulates numerous aspects of asexual and sexual growth and development (3, 7–16). *N. crassa* possesses three Gα, two Gβ, and one Gγ subunits (7–9, 13, 17–19), 45 predicted GPCRs (12, 15, 20) and one RIC8 ortholog (21). Deletion of *N. crassa ric8* leads to severe defects, including delayed spore germination, greatly reduced hyphal growth rate, female sterility, and aberrant asexual spore (macroconidia) development (21, 22). Levels of five G protein subunits are reduced in Δ*ric8* mutants, and *in vitro* GEF activity assays showed that RIC8 stimulates exchange of GDP for GTP on GNA-1 and GNA-3 in *N. crassa* (21).

In submerged shaking liquid cultures, wild-type *N. crassa* maintains hyphal growth. However, increased oxygen availability or exposure to stresses, such as heat, carbon, or nitrogen starvation, leads to formation of conidiophores and expression of conidiation-specific genes, including *con-10* (23–28). We have previously shown that Δ*gna-1* mutants produce hyphae in submerged liquid cultures when inoculated at 1 × 10^6^ cells/mL, but form conidiophores when inoculated at 3 × 10^6^ cells/mL (29). In the Δ*gna-3* mutant, inappropriate conidiation and *con-10* expression are observed in submerged cultures, even when inoculated at 1 × 10^6^ cells/mL (18). This phenotype and expression of *con-10* isare also observed in strains mutated in the adenylyl cyclase gene *cr-1* at 1 × 10^6^ cells/mL (18). Furthermore, when *gna-1* is deleted in addition to *gna-3*, there is an increase in the production of conidiophores and expression of the *con-10* gene at 1 × 10^6^ cells/mL (10). The Δ*ric8* strain produces conidiophores in submerged cultures at levels like that of a Δ*gna-1*, Δ*gna-3* double mutant (10, 21). The observation that the addition of 2% peptone to the medium suppresses conidiation, and the expression of *con-10* in Δ*gna-3*, Δ*gna-1* Δ*gna-3*, Δ*gna-2* Δ*gna-3,* and triple Gα mutants suggests that the submerged conidiation phenotype of these mutants stems from a metabolic defect (10). The effect of peptone has also been observed for other *N. crassa* mutations that influence nutrient sensing or signaling, such as loss of the predicted glucose sensor *rco-3* or the adenylyl cyclase *cr-1* (18, 28).

To investigate possible links between metabolism and development, we previously used ^1^H nuclear magnetic resonance to profile metabolites from wild type and the Δ*gna-3* mutant cultured in low and high carbon (30). This study showed that the global metabolome of the Δ*gna-3* mutant grown under either condition was like that of wild type cultured on high carbon, supporting a carbon-sensing defect in the Δ*gna-3* mutant under low carbon conditions (30). However, to date, metabolomics studies have not been performed with other mutants lacking genes implicated in G protein signaling in *N. crassa*.

In this study, RNA sequencing and targeted metabolomics were used to determine gene expression and metabolic profiles in wild-type and three G protein-signaling mutants (Table 1) cultured in a medium with 100 mM glucose, using an inoculation density of 1 × 10^6^ cells/mL. We analyzed two strains that contain only hyphae in submerged liquid cultures under this condition (wild-type and Δ*gna-1* strains) and two that produce hyphae and conidia (Δ*gna-3* and Δ*ric8* mutants). The results support links between the submerged conidiation defect of the Δ*ric8* mutant and defects in energy metabolism and levels of arginine and ornithine.

**TABLE 1:** Strains used in this study

Relevant genotype^*a*^	Strain name	Detailed genotype	NCU number(s)^*c*^	Source or reference^*b*^
Wild type	74-OR23-1A (FGSC2489)	Wild type, *mat A*	−	FGSC
∆*gna-1*	3b10	∆*gna-1::hph*, *mat a*	NCU06493	(31)
∆*gna-3*	31c2	∆*gna-3::hph*, *mat A*	NCU05206	(18)
∆*ric8*	R81-5a	∆*ric8::hph*, *mat a*	NCU02788	(21)
∆*arg-14*	FGSC22198	∆*arg-14::hph*, *mat a*	NCU07682	FGSC
∆*gpr-5*	#53-1	∆*gpr-5::hph*, *mat A*	NCU00300	(20)
∆*gpr-6*	#61-9	∆*gpr-6::hph*, *mat A*	NCU09195	(20)
∆*gpr-5,* ∆*gpr-6*	5,6#2	∆*gpr-5::hph*, ∆*gpr-6::hph*, *mat A*	NCU00300, NCU09195	This study
∆*vsb-1*	FGSC19508	∆*vsb-1::hph*, *mat a*	NCU02632	FGSC

^*a*^ The ortholog of NCU02632 is known as vacuolar storage of basic amino acids 1 (*VSB1*) in *Saccharomyces cerevisiae*. The *N. crassa* gene was previously mis-annotated as a sulfate transporter (*cys-23*). Based on the identified phenotypes in this study, we have adopted *vsb-1* as the name for the *N. crassa* gene.

^*b*^ FGSC, Fungal Genetics Stock Center.

^*c*^ NCU number(s), *Neurospora crassa* gene number(s).

## RESULTS

### Δ*gna-3* and Δ*ric8* mutants share the greatest number of differentially expressed genes

**Fig 1:**
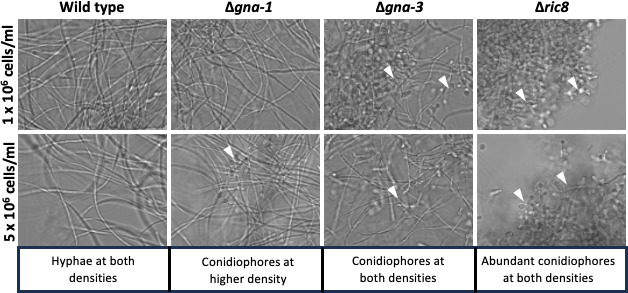
Morphology of strains in liquid submerged cultures. The indicated strains (see Table 1) were grown in Vogel’s minimal medium (VM) + glucose liquid medium for 16 hours as described in Materials and Methods. Samples were viewed using an Olympus IX71 inverted microscope with a 40× oil immersion objective, and images were captured using a QIClickTM digital CCD camera. The initial inoculum cell density for cultures is shown on the left side of the figure. White arrows indicate conidiophores. A summary of the observed morphology (hyphae vs conidiophores) is provided below each group of panels.

**Fig 2:**
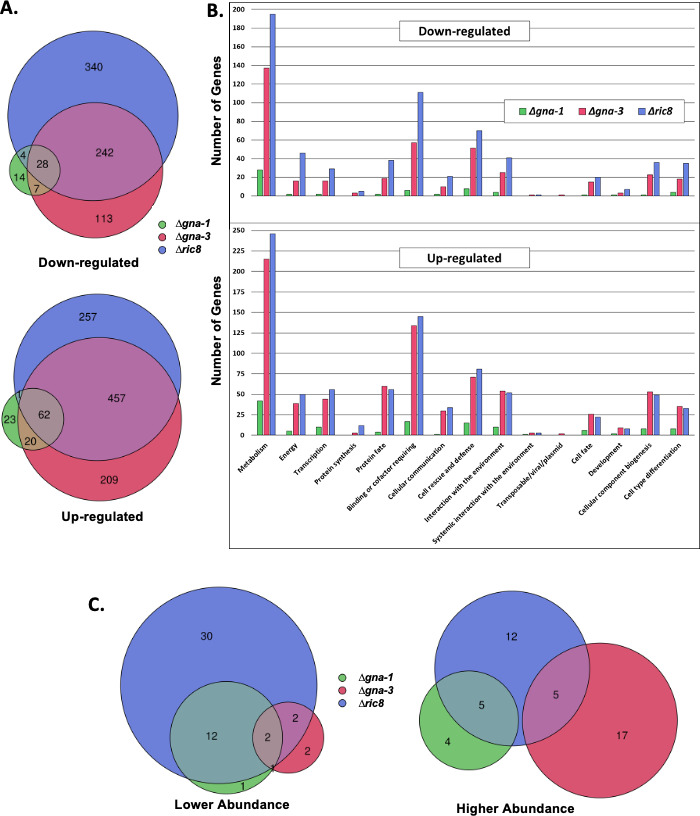
Differentially expressed genes and relative abundances of metabolites in the three mutants. (A) Differentially expressed genes identified during RNAseq analysis. Genes with >10 transcripts per million (TPMs) for all strains and that differed significantly from wild type with a fold change of ≥2.0 were included. The numbers in the lobes of the two Venn diagrams represent the number of shared genes that are either down (top) or up (bottom) regulated in one, two, or all three mutants as compared to wild type. (B) Functional catalog (FunCat) analysis. The distribution of FunCat assignments for all genes differentially regulated in the Δ*gna-1*, Δ*gna-3*, and Δ*ric8* mutants relative to wild type is shown. Downregulated (top) and upregulated (bottom) genes are included. (C) Relative abundances of metabolites determined using liquid chromatography-mass spectrometry (LC-MS). Metabolites with significantly different levels in the mutants vs wild type were determined using Student’s *t*-test. The numbers in the lobes of the two Venn diagrams represent the number of metabolites that are either present at lower (left) or higher (right) abundance in one, two, or all three mutants as compared to wild type.

As previously reported, microscopic analysis demonstrated that wild-type and Δ*gna-1* strains form only hyphae at an inoculation density of 1 × 10^6^ cells/mL in liquid cultures (Fig. 1). At an inoculation density of 5 × 10^6^, wild-type strains only produce hyphae, while conidiophores are visible in Δ*gna-1* strains (Fig. 1). In contrast, Δ*gna-3* and Δ*ric8* strains form abundant conidiophores at both inoculation densities, with Δ*ric8* strains having the more severe phenotype (Fig. 1). To identify mis-regulated genes that might form the basis for the morphological phenotypes, the transcriptomes of wild type, Δ*gna-1*, Δ*gna-3*, and Δ*ric8* strains were determined using poly-A mRNAseq (File S1). The biological replicates for each strain showed good agreement after principal component analysis (PCA; Fig. S1). In the Δ*gna-1* mutant, a relatively modest number (159 total) of differentially expressed genes were observed, with 53 downregulated and 106 upregulated (Fig. 2A). The Δ*gna-3* strain showed greater differences, having 390 genes downregulated and 748 upregulated, for a total of 1,138 mis-regulated genes. Δ*ric8* mutants exhibited the largest difference compared to wild type, with 614 downregulated and 777 upregulated genes, for a total of 1,391 genes (Fig. 2A). There was significant overlap in mis-regulated genes between Δ*gna-3* and Δ*ric8* mutants, but very few genes shared between Δ*gna-1* and the other two mutants (Fig. 2A). Like the trend noted in the total number of differentially expressed genes, 597 genes were uniquely mis-regulated in the Δ*ric8* mutant and 322 and 37 in the Δ*gna-3* and Δ*gna-1* strains, respectively (Fig. 2A).

### Many differentially regulated genes have predicted metabolic or conidiation functions

Functional catalog (FunCat) analysis was performed to investigate the distribution of predicted functions for the mis-regulated genes in each mutant (Fig. 2B). The functional category that was most highly represented in all three mutant strains was “metabolism,” and roughly one-third of the metabolic genes possessed an Enzyme Commission (32) number. In terms of total numbers of genes, the Δ*ric8* mutant has the most in each functional category, followed by the Δ*gna-3* and then Δ*gna-1* strains, which tracks the total number of differentially regulated genes in each mutant for downregulated genes. However, there are five upregulated categories in which the Δ*gna-3* mutant possesses the most genes: “protein fate,” “interaction with the environment,” “cell fate,” “cellular component biogenesis,” and “cell type differentiation” (Fig. 2B).

The mitochondrial electron transport chain of *N. crassa* contains several major complexes that couple oxidation reactions to proton translocation to power ATP generation (33, 34). Complex I is composed of 32 nuclear-encoded and seven mitochondrially encoded proteins in *N. crassa* (35). Of the nuclear-encoded Complex I transcripts, 21 were significantly downregulated in the Δ*ric8* mutant (File S2), with an average reduction of 2.4-fold (Table 2). Expression of most components in Complexes II, III, and IV was like wild type for all mutants, with the exception of NCU01808 (*cyc-1*) from Complex III, which was upregulated 2.7-fold in the Δ*ric8* mutant (Table 2). In addition, *aod-1* was upregulated in both the Δ*gna-3* and the Δ*ric8* mutants. AOD-1 transfers electrons from the ubiquinol pool directly to oxygen (36), thus providing a bypass for strains with deficiencies in Complexes III and IV of the electron transport system (ETS).

**TABLE 2:** Expression of several electron transport chain and conidiation genes relative to wild type

Gene class	Gene number	Gene name	Fold change^*a*^		
			Δ*gna-1*	Δ*gna-3*	Δ*ric8*
Electron transport chain genes					
Complex I	NCU11348	*nuo11.3*	1.0	−1.5	**−2.8**
	NCU01467	*nuo10.4*	1.0	−1.6	**−2.6**
	NCU01360	*nuo11.5*	1.0	−1.4	**−2.5**
	NCU03093	*nuo12.3*	1.0	−1.5	**−2.7**
	NCU09299	*nuo14*	1.0	−1.3	**−2.1**
	NCU00484	*nuo18.4*	1.0	−1.4	**−2.3**
	NCU02472	*nuo20.8*	1.0	−1.6	**−2.3**
	NCU01859	*nuo20.9*	1.0	−1.5	**−2.2**
	NCU05221	*nuo21*	1.0	−1.4	**−2.6**
	NCU08930	*nuo21.3a*	1.0	−1.3	**−2.6**
	NCU02280	*nuo21.3b*	1.0	−1.4	**−2.4**
	NCU05009	*nuo21.3c*	1.0	−1.5	**−2.5**
	NCU01169	*nuo24*	1.0	−1.2	**−2.0**
	NCU05299	*nuo29.9*	1.0	−1.4	**−2.9**
	NCU04074	*nuo30.4*	1.0	−1.4	**−2.3**
	NCU02534	*nuo49*	1.0	−1.5	**−2.4**
	NCU04044	*nuo51*	1.0	−1.2	**−2.0**
	NCU00160	*nuo6.6*	1.0	−1.5	**−2.4**
	NCU01765	*nuo78*	1.0	−1.3	**−2.3**
	NCU00670	*nuo9.5*	1.0	−1.7	**−2.8**
	NCU04781	*nuo9.8*	1.0	−1.5	**−2.6**
Complex IV	NCU01808	*cyc-1*	1.0	1.4	**2.7**
Alternative oxidase	NCU07953	*aod-1*	1.0	**4.5**	**16.3**
Conidiation-related genes					
Aerial hyphae	NCU06799	*vad-5*	1.5	1.4	1.0
	NCU01713	*ve-1*	1.0	1.0	1.0
Minor constrictions	NCU00478	*acon-2*	1.4	1.3	1.4
	NCU09739	*fld*	1.8	1.97	1.0
Major constrictions	NCU07617	*acon-3*	**39.8**	**148.9**	**21.1**
	NCU08726	*fl*	**2.5**	**10.4**	**4.8**
Conidial release	NCU02713	*csp-1*	1.9	**6.1**	**6.8**
	NCU06095	*csp-2*	1.3	**8.7**	**5.5**
*fl* regulation	NCU03725	*vib-1*	−1.4	−1.9	−1.9
	NCU03043	*flb-3*	1.3	**3.7**	**2.7**
Conidiation-regulated genes	NCU08769	*con-6*	NE^*b*^	**1584.4**	**7585.2**
	NCU07325	*con-10*	NE	**347.2**	**1912.6**
	NCU07324	*con-13*	**76.7**	**7344.5**	**3258.8**

^*a*^ Positive numbers indicate increased expression in the mutant, while negative numbers indicate decreased expression. Boldface font indicates fold change regulation 2-fold or greater.

^*b*^ NE, genes that are not expressed.

Since at lower inoculation densities, Δ*gna-3* and Δ*ric8* inappropriately form conidiophores in liquid culture, and at higher cell densities, Δ*gna-1* strains will also produce some conidiophores (Fig. 1), we inspected our data for differences in the expression of several genes that are associated with macroconidia production (37) (File S2; Table 2). None of the mutants mis-regulated *vad-5*, *ve-1*, *acon-2*, and *fld*, which are involved in aerial hyphae production and formation of minor constrictions in developing conidiophores. In contrast, two genes required for the formation of major constrictions, *acon-3* and *fl*, were upregulated in all three mutants to varying degrees, with Δ*gna-3* the most affected (Table 2). The *csp-1* and *csp-2* genes are involved in conidial release. They were only upregulated in Δ*gna-3* and Δ*ric8* mutants (Table 2), which may explain the tendency for these strains to produce mature conidia in submerged cultures.

When overexpressed, the *fl* (*fluffy*) gene is sufficient to cause conidial development in submerged cultures (38). We observed increased *fl* expression in all three mutants, yet Δ*gna-1* mutants do not exhibit inappropriate conidiation unless inoculated at a higher cell density. This may be explained by the differing levels of *fl* upregulation: 2.5-, 10.4-, and 4.8-fold increases in Δ*gna-1*, Δ*gna-3*, and Δ*ric8* mutants, respectively (Table 2). Expression of the *con-6* and *con-10* genes is elevated when *fl* is overexpressed (39). However, the mutant with the highest expression of *con-6* and *con-10* is Δ*ric8*, which has lower levels of *fl* transcript than Δ*gna-3*. The *con-6* and *con-10* genes contain several elements in their promoters that are regulated by development, light, and the circadian clock (37). Thus, the higher expression level of these genes in the Δ*ric8* mutant may result from defects in multiple transcriptional regulatory input systems.

The target of rapamycin (TOR) kinase and AMP kinase (AMPK) signaling pathways are crucial for metabolic regulation in eukaryotes (40–43). TOR activation is associated with anabolic processes and cell growth, while AMPK negatively regulates TOR to activate autophagy and catabolism (42). In addition, recent work in *N. crassa* has linked regulation of the conidiation rhythm to TOR signaling (43–45). We interrogated our RNAseq data set for differential expression of genes implicated in TOR and AMPK signaling (File S2). Out of 37 genes, we observed only two that met our cut-off; the transcription factor *ada-16*, homologous to *Saccharomyces cerevisiae* SFP1, was upregulated 2.24-fold in the Δ*ric8* mutant, and *stk-11*, homologous to the ribosomal protein S6 kinase, was upregulated twofold in the Δ*gna-3* mutant. Although Sfp1p positively regulates transcription of ribosomal protein-encoding genes in yeast (46), no ribosomal protein-coding genes were differentially regulated in any mutant (File S2).

### The metabolome of the Δ*ric8* mutant exhibits the most differences relative to wild type

Having observed that many of the differentially expressed genes in the mutants were implicated in metabolism, we implemented targeted liquid chromatography-mass spectrometry (LC-MS) metabolomics to determine whether the effect on transcription was reflected in changes to metabolite levels. A total of 201 standards were used to detect metabolites, with 121 metabolites unambiguously identified in all four strains (File S3). PCA with three dimensions was conducted to represent metabolite differences between the different genotypes (Fig. S2). Following the same pattern as the RNAseq, the Δ*gna-1* mutant had the lowest number of mis-regulated metabolites relative to wild type, with 25, followed by the Δ*gna-3* and Δ*ric8* strains, with 29 and 68, respectively (Fig. 2C). The most represented category among the mis-regulated metabolites in all mutants was amino acids (Table S1). In contrast to the results from the RNAseq analysis, Δ*gna-1* and Δ*ric8* mutants shared the most mis-regulated metabolites, with 19, followed by Δ*ric8* and Δ*gna-3* mutants with nine metabolites (Fig. 2C).

### Evidence for transcriptional regulation influencing tryptophan and energy metabolism

We next compared the results from RNAseq and metabolomics analysis to identify cases of probable transcriptional control of metabolic enzymes. Tryptophan was present in higher amounts in the Δ*gna-3* mutant (File S2). The transcript for tryptophan 2,3-dioxygenase (*nt*; NCU05752), encoding the first enzyme in the kynurenine pathway that catabolizes tryptophan to produce NAD^+^ (47, 48), was downregulated in all three mutants, with the greatest decrease in the Δ*gna-3* strain (File S2). Lower levels of the enzyme may lead to the accumulation of tryptophan in the Δ*gna-3* mutant.

As mentioned above, we noted decreased expression of several components of the ETS in the Δ*ric8* mutant (Table 2). NADH and NADPH were not assayed in our experiments, but relative levels of NAD^+^ were lower in the Δ*gna-3* and Δ*ric8* strains, while NADP^+^ was reduced in the ∆*gna-1* and ∆*ric8* mutants (File S3). *utr-1* (NCU03267), encoding NAD^+^ kinase, the enzyme that converts NAD^+^ to NADP^+^, was expressed to higher levels in the ∆*ric8* and ∆*gna-3* strains (File S2). UTR-1 regulates redox homeostasis in many organisms (49), and its elevated expression in the two mutants that are producing conidia may reflect a hyperoxidant state in these strains. In contrast, *nic-11* (NCU01140), encoding nicotinamide nucleotide transhydrogenase, the enzyme that interconverts NAD^+^ and NADP^+^, is downregulated relative to wild type in the ∆*ric8* and ∆*gna-3* strains (File S2). This finding further supports a problem with interconversion of NAD^+^ and NADP^+^ in the two mutants, which is more severe in the ∆*ric8* strain. Δ*ric8* mutants had lower levels of all three adenosine phosphate metabolites (ATP, ADP, and AMP; File S3). These observations suggest systematic issues in the ETS stemming from downregulation of Complex I.

### Arginine metabolism is significantly perturbed in Δ*ric8* mutants

**Fig 3:**
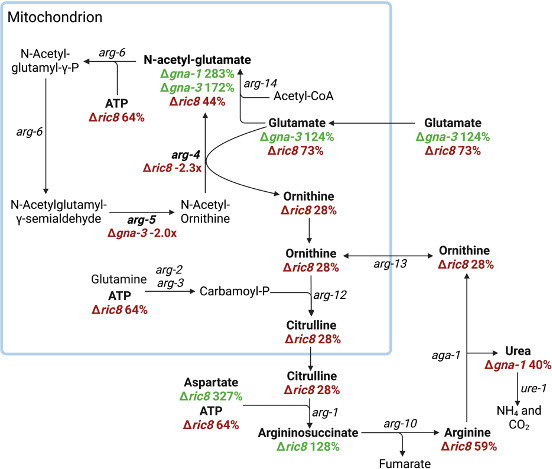
Arginine metabolism is altered in the Δ*gna-1*, Δ*gna-3*, and Δ*ric8* mutants. Green text indicates elevated levels, and red text indicates reduced levels of transcripts or metabolites. Genes encoding arginine metabolic enzymes are indicated in italic text. Genes that are differentially expressed in a mutant relative to wild type are indicated, with the fold change displayed below the gene name. Metabolites detected by LC-MS are bolded. The % indicates metabolites with significantly different relative levels (as determined by Student’s *t*-test) in the mutants compared to wild type. *arg-14* = NCU07682, acetylglutamate synthase; *arg-4* = NCU10468, N-acetylornithine-glutamate acetyl transferase; *arg-5* = NCU05410, acetylornithine aminotransferase; *arg-6* = NCU00567, acetylglutamate kinase; *arg-2* = NCU07732, carbamoyl-phosphate synthase small subunit; *arg-3* = NCU02677, carbamoyl-phosphate synthase large subunit; *arg-12* = NCU01667, ornithine transcarbamylase; *arg-1* = NCU02639, argininosuccinate synthetase; *arg-10* = NCU08162, argininosuccinate lyase; *aga-1* = NCU02333, arginase; *arg-13* = NCU02802, mitochondrial ornithine carrier. Figure was created using biorender.com.

We noted that levels of several compounds involved in arginine metabolism were altered in some of the mutants. In Δ*gna-1* strains, N-acetylglutamic acid (234% of wild type) and urea (40% of wild type) are mis-regulated (Fig. 3; File S3). These compounds are involved in arginine biosynthesis (N-acetylglutamic acid) or catabolism (urea). However, there were no differentially expressed arginine pathway transcripts in the Δ*gna-1* mutant (File S2), suggesting possible post-transcriptional regulation. Although the Δ*gna-3* mutant had reduced expression of *arg-5* (NCU05410; File S2), arginine levels were normal, and glutamate (124%) and N-acetylglutamate (172%) amounts were actually elevated (Fig. 3; File S3). The relative amounts of several metabolites involved in arginine biosynthesis were reduced in the Δ*ric8* mutant relative to wild type, including N-acetylglutamate (44%), glutamate (73%), ornithine (28%), citrulline (28%), ATP (64%), and arginine (59%; Fig. 3). In contrast, levels of two other metabolites were elevated: argininosuccinate (135%) and aspartate (327%) (Fig. 3; File S3). Of these six compounds, there was evidence for transcriptional control in two cases: aspartate and N-acetylglutamate. *hom-1* (NCU00554), encoding the enzyme that initiates the conversion of aspartate into homoserine, and *arg-4* (NCU10468), encoding the enzyme that converts glutamate and N-acetylornithine into ornithine and N-acetylglutamate, were both downregulated in the Δ*ric8* mutant (File S2).

Based on the evidence for possible transcriptional or post-transcriptional control of arginine pathway metabolites in some mutants, we performed enzymatic activity assays for the pathway enzymes in cases where either a substrate or product intermediate had a different relative level compared to wild type. In the ∆*gna-1* mutant, we measured the activity of N-acetylornithine-glutamate acetyl transferase (50, 51) (ARG-4) and the catabolic enzyme arginase (52) (AGA-1). The activity of ARG-4 was reduced in the ∆*gna-1* mutant to 66% of wild type (Table 3), in opposition to the increased N-acetylglutamate levels (Fig. 3). In contrast, the activity of AGA-1 in the ∆*gna-1* strain was 66% of wild type (Table 3), which correlates with the reduced amount of urea (Fig. 3). We also measured the activity of N-acetylornithine-glutamate acetyl transferase/ARG-4 in the ∆*gna-3* mutant, with the results showing a significant reduction in activity to 51% of wild type (Table 3). However, like the case for the ∆*gna-1* mutant, the reduction in ARG-4 activity did not correlate with the elevated N-acetylglutamate levels in the ∆*gna-3* strain (Fig. 3). Although *arg-5* was differentially expressed in the ∆*gna-3* mutant, the enzyme was not tested for activity because the major precursor and product metabolites were not assayed in our study (Fig. 3).

**TABLE 3:** Activity of arginine metabolic enzymes in the three mutants

Enzyme	Strain genotype	% Wild-type activity^*a*^	SE	*P*-value^*b*^
N-acetylornithine-glutamate acetyl transferase (ARG-4)	∆*gna-1*	66.4	0.0263	1.82 × 10^−7^
	∆*gna-3*	50.7	0.0464	3.65 × 10^−6^
	∆*ric8*	50.7	0.0431	3.40 × 10^−6^
Ornithine transcarbamylase (ARG-12)	∆*ric8*	99.0	0.00812	0.655
Argininosuccinate synthetase (ARG-1)	∆*ric8*	60.9	0.00307	0.000696
Argininosuccinate lyase (ARG-10)	∆*ric8*	80.4	0.0393	0.0187
Arginase (AGA-1)	∆*gna-1*	65.9	0.0367	0.00107
	∆*ric8*	76.2	0.0295	0.00192

^*a*^ Activity was measured as the nmoles of product produced per time microgram cell extract protein. The values for each mutant were normalized to wild type and expressed as a %.

^*b*^ Compared to wild type; determined using Students *t*-test.

In the ∆*ric8* mutant, we assayed five enzymes: N-acetylornithine-glutamate acetyl transferase/ARG-4, ornithine transcarbamylase (OTC; ARG-12) (53), argininosuccinate synthase (ASS; ARG-1) (53), argininosuccinate lyase (ASL; ARG-10) (53), and arginase/AGA-1 (Fig. 3) (52). ARG-4 activity was reduced to 51% of wild type, which correlates well with the decreased expression of *arg-4* and lower levels of N-acetylglutamate and ornithine in the ∆*ric8* mutant, consistent with transcriptional control (Table 3). The activity of OTC in the Δ*ric8* mutant was similar to wild type; since the levels of the substrate ornithine and product citrulline were reduced identically, there is no evidence for post-transcriptional regulation (Table 3). Based on the higher relative levels or argininosuccinate and aspartate and the lower relative levels of arginine, ornithine, and citrulline in the Δ*ric8* mutant strain, we hypothesized that the Δ*ric8* mutant should have increased ASS activity relative to wild type. In contrast, the activity of ASS in the Δ*ric8* mutant was 61% of wild type, while ASL activity was 80% of wild type (Table 3), consistent with a slight reduction in the formation of arginine from argininosuccinate in the Δ*ric8* mutant. Finally, the AGA activity in the Δ*ric8* mutant was 76% of wild type, which should lead to slightly increased arginine and decreased ornithine levels. However, arginine levels were reduced in the mutant (Table 3).

The elevated levels of N-acetylglutamate could not be explained by the activity of ARG-4 in the ∆*gna-1* and ∆*gna-3* mutant strains. However, N-acetylglutamate is also produced by the activity of acetylglutamate synthase (ARG-14; NCU07682; Fig. 3) (54). The *arg-14* gene is not differentially expressed in any of the mutant strains (File S2). To investigate possible post-transcriptional regulation of ARG-14 levels, we performed western analysis using an antibody against the ARG-14 protein (55). The results showed that a protein corresponding to the reported size of ARG-14 (~70 kDa) (55) is present at similar levels in wild type and the three mutants and absent from the ∆*arg-14* strain (Fig. S4), not explaining the elevated N-acetylglutamate levels observed in the ∆*gna-1* and ∆*gna-3* mutants. Therefore, the activity and levels of the arginine metabolic enzymes did not account for the elevated amount of N-acetylglutamate in the ∆*gna-1* and ∆*gna-3* mutants or the low levels of arginine, ornithine, and citrulline in the Δ*ric8* mutant.

### Expression of two predicted vacuolar arginine transporter genes is altered in the Δ*ric8* mutant

Since the results from analysis of arginine pathway enzymes did not provide a complete explanation for the metabolic defects in the Δ*ric8* mutant, we explored other possibilities. The levels of amino acids in hyphae and macroconidia have been determined, with levels of arginine, ornithine, and citrulline lower in macroconidia than in hyphal cultures (30, 56). We again measured levels of arginine and ornithine and observed low amounts in macroconidia compared to hyphae in wild type (Table S2). The finding that the Δ*ric8* mutant inappropriately produces asexual spores in submerged culture (Fig. 1) (21) suggested that the low levels of arginine and ornithine may result from or lead to the formation of macroconidia by this mutant.

**Fig 4:**
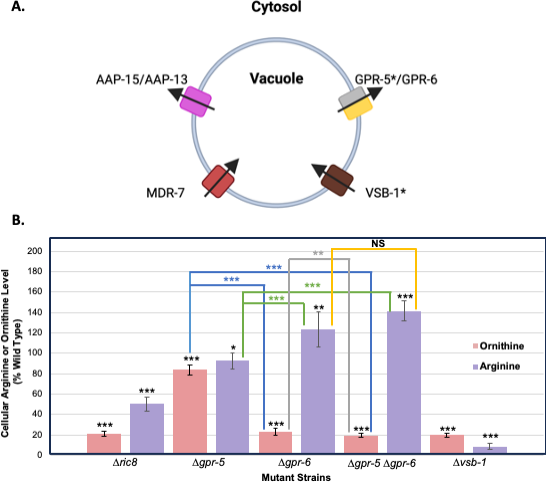
Levels of arginine and ornithine in vacuolar transporter mutants. (A) Schematic showing the action of predicted vacuolar arginine transporters. The predicted direction of arginine transport by *N. crassa* proteins identified as orthologs of *S. cerevisiae* vacuolar transporters is shown using arrows. Asterisks indicate genes that were differentially expressed in the Δ*ric8* mutant relative to wild type. Figure was created using biorender.com. (B) Total arginine and ornithine pools. Strains were grown, metabolites were extracted and separated using ion exchange chromatography, and arginine and ornithine levels were determined as described in Materials and Methods. Amino acid levels were normalized to the dry weight of the samples. Significant differences relative to wild type are represented using black asterisks, while differences relative to other mutants are shown using colored bars and asterisks (*, *P* < 0.05; **, *P* < 0.01; ***, *P* < 0.001).

In *N. crassa*, the majority (98%–99%) of the cellular pools of arginine and ornithine are sequestered within the vacuole (50, 53). Nitrogen starvation results in movement of stored amino acids into the cytoplasm, where several enzymes catabolize arginine and ornithine to form glutamate, proline, and ammonium, which are further converted into other molecules to support cell growth (53). We hypothesized that arginine and ornithine are similarly expelled from the vacuole during macroconidiation, leading to their degradation in the cytoplasm, and expression of vacuolar transporters may be altered in wild type during macroconidiation and in the Δ*ric8* mutant in submerged cultures. We identified six *N. crassa* orthologs of genes that influence vacuolar arginine transport in *S. cerevisiae* (Table 4; Fig. 4A). Of these, one was downregulated (*vsb-1*: NCU02632), and one was upregulated (*gpr-5*: NCU00300) in the Δ*ric8* mutant (File S2). Deletion of *VSB1* results in arginine exclusion from the vacuole in *S. cerevisiae* and Vsb1p is considered the major transporter responsible for vacuolar arginine retention in yeast (57). Reduced expression of *N. crassa vsb-1* in the ∆*ric8* mutant is consistent with the eviction of arginine from the vacuole and its catabolism in the cytosol. Transporter opsin GPCR (TOG) family transporters YPQ1/2/3 are variously required for release of arginine from the vacuole in *S. cerevisiae* (57). We observed upregulation of *YPQ1/3* ortholog *gpr-5* in the Δ*ric8* mutant, consistent with lower arginine levels.

**TABLE 4:** *N. crassa* orthologs of genes involved in vacuolar transport of arginine in *S. cerevisiae*

Superfamily	*N. crassa* gene	*S. cerevisiae* gene	Direction	Characterized function(s) in *S. cerevisiae*	Reference
Amino acid polyamine organocation	*vsb-1^a^* NCU02632	*VSB1*	In	Vacuolar membrane protein necessary for the uptake of arginine into the vacuole in minimal medium. Mutant has low arginine levels in minimal medium.	(57)
TOG	*gpr-5* NCU00300	*YPQ1* and *YPQ3^b^*	Out	PQ-loop vacuolar membrane proteins required for arginine transport out of the vacuole during nitrogen starvation and overall increase in arginine levels. Single mutants have normal arginine levels in minimal medium.	(57)
	*gpr-6* NCU09195	*YPQ1* and *YPQ3*	Out		
Amino acid auxin permease	*aap-15* NCU03783	*AVT3* and *AVT4*	Out	Basic and neutral amino acid AAP family exporters in the vacuolar membrane that function mainly during nitrogen starvation. Mutation of *AVT4* (but not *AVT3*) leads to slightly higher arginine levels in nitrogen-replete medium.	(58)
	*aap-13* NCU05775	*AVT3* and *AVT4*	Out		
MFS (major facilitator superfamily)	*mdr-7* NCU01095	*VBA2*	In	Basic amino acid importer in the vacuolar membrane. Vacuoles from the mutant have a defect in arginine uptake.	(59)

^*a*^ Previously named *cys-23*; we have adopted the yeast name *vsb-1* for NCU02632 based on phenotypes determined during this study.

^*b*^ *RCT2* and *YPQ3* are alternate names for the same gene in *S. cerevisiae*.

We also investigated our RNAseq data set for the expression of other genes that influence transport of basic amino acids, including the regulatory and structural subunits of the vacuolar ATPase (60), as well as plasma membrane arginine transporters; however, none were differentially expressed using the twofold threshold (File S2). Expression of the mitochondrial ornithine transporter, *arg-13* (61–63), was increased 1.9-fold in the ∆*ric8* mutant (File S2), consistent with an increased movement of ornithine into the cytoplasm. However, this is not predicted to have a pronounced effect on the cellular ornithine level, as only 1% of the pool is contained in mitochondria (61, 64).

### Several vacuolar transporter mutants possess altered levels of arginine and ornithine

The possibility of altered compartmentation due to dysregulation of transport genes in the Δ*ric8* mutant led us to measure total cellular pools of arginine and ornithine in wild type, Δ*ric8*, Δ*vsb-1*, and Δ*gpr-*5 strains (Fig. 4B). Although *gpr-6* (NCU09195) was not mis-regulated in the Δ*ric8* mutant, we also included the Δ*gpr-6* single and Δ*gpr-5* Δ*gpr-6* double mutants, as *gpr-5* and *gpr-6* share significant amino acid similarity in *N. crassa* (20). The pools of arginine and ornithine in the Δ*ric8* mutant were in good agreement with the results from LC-MS, at 50% and 21% of wild type, respectively (Fig. 4B). The Δ*vsb-1* mutant had very low levels of arginine (9%) and ornithine (20%) compared to wild type (Fig. 4B), consistent with the phenotype of the yeast mutant (57), strongly supporting an important role in arginine and ornithine retention in the vacuole in *N. crassa*. In the Δ*gpr-5* mutant, arginine and ornithine levels showed a modest reduction relative to the wild-type strain (92% and 83%; Fig. 4B). In the Δ*gpr-6* strain, the amounts of arginine and ornithine exhibited an inverse relationship, with ornithine lower and arginine higher than in wild type (23% and 123%, respectively; Fig. 4B). The Δ*gpr-5* Δ*gpr-6* double mutant had elevated arginine levels like the single Δ*gpr-6* mutant, but ornithine was slightly more reduced (Fig. 4B). The increased arginine amount in single and double mutants lacking *gpr-6* in *N. crassa* is consistent with negative regulation of arginine retention by GPR-6. In contrast, the results suggest that GPR-5 and GPR-6 positively regulate ornithine retention, with GPR-6 having a greater impact (Fig. 4B).

**Fig 5:**
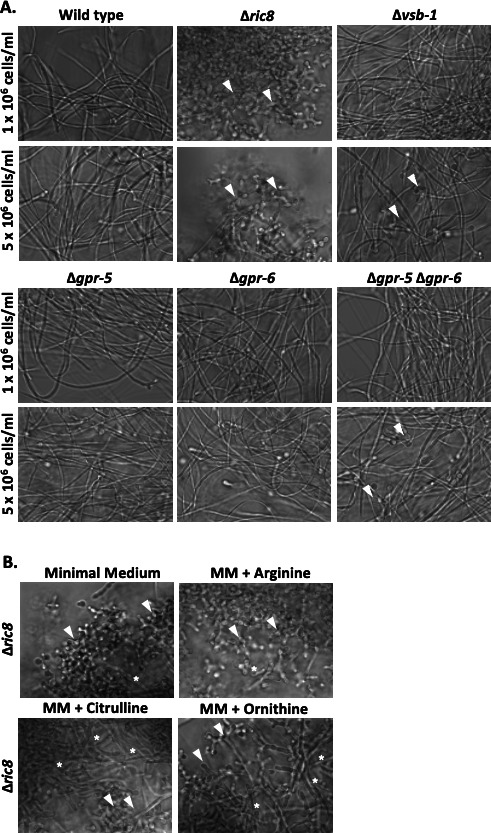
Impact of vacuolar transporter mutations and amino acid supplementation on strain morphology. (A) Morphology of vacuolar transporter mutants in liquid culture. Strains were inoculated in VM-Glucose at the two indicated cell densities (along left side of figure) and imaged as described in the legend to Fig. 1. White arrowheads indicate conidiophores. (B) Morphology of the Δ*ric8* mutant after amino acid supplementation. The Δ*ric8* strain was inoculated at a cell density of 1 × 10^6^ cells/mL and then grown in VM-Glucose minimal medium alone or also containing 100 µg/mL of arginine, ornithine, or citrulline. Cultures were imaged as described in the legend to Fig. 1. White arrows denote conidiophores, while asterisks indicate hyphae. MM = VM minimal medium.

The impact on arginine and ornithine pools in mutants lacking *ric8*, *vsb-1*, *gpr-5*, and/or *gpr-6*, prompted us to check the submerged culture morphology of these strains (Fig. 5A). The Δ*vsb-1* single and Δ*gpr-5* Δ*gpr-6* double mutants formed conidiophores at 5 × 10^6^ cells/mL, but arginine levels were lower and higher in the Δ*vsb-1* and Δ*gpr-5* Δ*gpr-6* mutants, respectively. There was a better correlation between conidiation and ornithine, as levels are lower in the two mutants (Fig. 5A). Finally, we attempted to correct the submerged conidiation phenotype in the Δ*ric8* mutant by supplementing with arginine, ornithine, and citrulline. Ornithine or citrulline resulted in a slight reduction in the phenotype (Fig. 5B). Based on the link between GNA-1 and GNA-3 with adenylyl cyclase and cyclic adenosine monophosphate (cAMP) signaling (18, 31, 65), we also tested the effect of cAMP. However, supplementation with cAMP alone or in combination with ornithine or citrulline did not provide additional correction of the phenotype.

## DISCUSSION

We began our study with the goal of identifying genes that were differentially expressed in mutants lacking the *ric8* GEF and two Gα subunits regulated by RIC8, *gna-1* and *gna-3*. These mutants have a morphological phenotype in that they produce macroconidia in submerged culture. We discovered that metabolic genes made up a large proportion of the differentially expressed transcripts and implemented LC-MS-targeted metabolomics to identify mis-regulated metabolites. These experiments enabled us to identify potential downstream metabolic pathways. We identified the greatest number of connections between metabolite levels and the submerged macroconidiation phenotype in the Δ*ric8* mutant, which also possesses the most severe defect of the three mutants in this study.

The observation of many downregulated transcripts for ETS Complex I proteins in the Δ*ric8* mutant suggests a defect in Complex I function (66). At the same time, the Δ*ric8* mutant has significantly elevated expression of cytochrome C (*cyc-1*) and alternative oxidase (*aod-1*). Intriguingly, it has been reported that a mutant with a defective Complex I gene (*nuo14*) exhibits upregulation of *cyc-1* and *aod-1* (66). The authors suggested that increased expression of alternative oxidase and cytochrome *c* may represent a general response to deficiencies in the mitochondrial respiratory chain (66). Our results with the Δ*ric8* mutant are consistent with this hypothesis.

Our data suggest that reduced expression of *arg-4* and *vsb-1* likely contributes to the low arginine levels observed in the Δ*ric8* mutant. ARG-4 (N-acetylornithine-glutamate acetyl transferase) is the entry point for most of the glutamate that enters the arginine biosynthetic pathway (53). Reduced expression of *arg-4* in the Δ*ric8* mutant would presumably increase reliance on the N-acetylglutamate synthase enzyme ARG-14, which is more expensive for the cell energetically (requires acetyl-S-CoA) (53). Our results also provide the first evidence that arginine and ornithine may use some of the same transporters in fungi. The Δ*vsb-1* mutant has low levels of both arginine and ornithine in *N. crassa*, suggesting that VSB-1 transports both amino acids into the vacuole. In contrast, ∆*gpr-6* mutants exhibit an inverse relationship for levels of arginine (higher) and ornithine (reduced), relative to wild type, possibly suggesting an antiporter relationship for arginine and ornithine.

We cannot rule out that the low levels of ATP in the ∆*ric8* mutant contribute to many of the metabolic differences and the submerged conidiation phenotype. Previous studies showed that the levels of ATP, ADP, and AMP are reduced in macroconidia compared to hyphal cultures (56, 67). Many metabolic reactions utilize the energy from ATP hydrolysis for synthesis of metabolites that may themselves be required for hyphal growth. Relevant to this, ATP is required for multiple steps in arginine biosynthesis (Fig. 3) (68) and for vacuolar arginine transport (69). The activity of VSB-1 is likely affected by low ATP levels in the ∆*ric8* mutant, as the yeast ortholog is dependent on the H^+^ gradient generated by the V-ATPase (57).

We propose that the submerged conidiation phenotype observed in the ∆*ric8* mutant is related to its low levels of ornithine, arginine, ATP, and other metabolites, reflective of an overall metabolic defect. The ∆*gna-3* mutant has a less severe submerged conidiation defect and also exhibits fewer metabolic differences relative to wild type, in spite of expressing the *fl* gene to the highest levels of the three mutants. One possibility is that the increased tryptophan and reduced NAD^+^ levels, resulting from downregulation of the tryptophan 2,3-dioxygenase transcript, are a trigger for submerged macroconidiation. Likewise, it is intriguing that the S6 kinase mRNA, encoding a TOR substrate, was uniquely upregulated in the Δ*gna-3* mutant, as a recent study reported a macroconidiation circadian rhythm in phosphorylation of the S6 ribosomal protein, which is itself a target of the S6 kinase, in *N. crassa* (43). Further work is necessary to determine whether the submerged conidiation defect of the ∆*gna-3* mutant is related to either or both of these possibilities.

Within cells, arginine has other vital functions beyond its role as a protein amino acid. Arginine is a precursor for the synthesis of other metabolites, including proline, ornithine, urea, glutamine, glutamate, polyamines, nitric oxide, and creatine (70). In contrast to baker’s yeast (41), there is some evidence that arginine may impact TOR signaling in *N. crassa* (44), and multiple mechanisms have been proposed for the action of arginine during TORC1 activation in humans (40). Future work in *N. crassa* will focus on possible roles for vacuolar compartmentation of amino acids and specific functions of arginine and ornithine in regulating growth and macroconidiation in *N. crassa*.

## MATERIALS AND METHODS

### Strains, media, and microscopy

The *N. crassa* strains used in this study are listed in Table 1. Conidia were propagated in Vogel’s minimal medium (VM) (71) agar flasks as described previously (72). Strains were grown in 50 mL of liquid VM, with 100 mM glucose as the carbon source (VM-Glucose). Where indicated, amino acids were present at 100 µg/mL. Liquid cultures were brought to a concentration of 1 × 10^6^ conidia/mL and incubated with shaking at 200 revolutions per minute (RPM) in the dark for 16 hours at 30°C, except when otherwise specified. Genotypes of mutants obtained during this study were validated using diagnostic PCR (Supplemental Methods). Cultures were imaged using differential interference contrast microscopy using an Olympus IX71 inverted microscope (Olympus America, Center Valley, PA) with a 40× oil immersion objective (NA  =  1.42). Images were captured using a QIClickTM digital CCD camera (QImaging, Surrey, British Columbia, Canada) and analyzed using Metamorph software (Molecular Devices Corporation, Sunnyvale, CA).

### Transcriptomics and metabolomics

Cultures were collected by vacuum filtration and stored at −80°C. Methods for tissue extraction, RNA isolation, library preparation, and downstream bioinformatics analysis of RNAseq data are described in the Supplemental Methods. Sample extraction and targeted analysis of polar, primary metabolites were performed at the UC Riverside Metabolomics Core Facility as previously described, using five biological replicates for each strain (73). Methods are summarized in the Supplemental Methods.

### Enzymatic assays, measurement of arginine and ornithine pools, and western analysis

For enzymatic assays, strains were grown under the same conditions used for the RNAseq and LC-MS samples, except that the culture volumes were 500 mL for wild type and 1 L for mutant strains. Details for sample lysis, enzyme activities, and measurement of arginine and ornithine levels are in the Supplemental Methods. For western analysis of ARG-14, strains were grown under the same conditions used for the RNAseq and LC-MS samples, while the ∆*arg-14* strain was cultured for 32 hours due to slow growth. Details for sample lysis and western blot analysis are in the Supplemental Methods.

## Data Availability

The data from this study are fully available without restrictions. All mRNAseq and metabolomics data, as well as several downstream processed files, are available in the supplemental files.

## SUPPLEMENTAL MATERIAL

File S1 mbio.03133-24-s0001.xlsx TPMs and global DEseq files.

File S2 mbio.03133-24-s0002.xlsx DEseq for specialized gene groups.

File S3 mbio.03133-24-s0003.xlsx Complete LC-MS data.

Supplemental material mbio.03133-24-s0004.pdf Details for some methods and as well as supplemental tables and figures.

ASM does not own the copyrights to Supplemental Material that may be linked to, or accessed through, an article. The authors have granted ASM a non-exclusive, world-wide license to publish the Supplemental Material files. Please contact the corresponding author directly for reuse.
