# Supplementary material for: RNAseq and targeted metabolomics implicate RIC8 in regulation of energy homeostasis, amino acid compartmentation, and asexual development in Neurospora crassa
Source: mBio. 2024 Nov 18;15(12):e03133-24. doi: 10.1128/mbio.03133-24 (PMC11633382; doi:10.1128/mbio.03133-24)
Supplement: Supplemental material — Details for some methods and as well as supplemental tables and figures. [file mbio.03133-24-s0004.pdf]

SUPPLEMENTAL METHODS, TABLES AND FIGURES

SUPPLEMENTAL METHODS

Purification of a homokaryotic Δ*arg-14* mutant and strain genotype validation of Δ*arg-14* and Δ*vsb-1* mutants. The *vsb-1*::*hph* (FGSC19508) and ∆*arg-14::hph* (FGSC22198) mutants were generated during the Neurospora knockout project (1). The *vsb-1*::*hph* strain was deposited as a homokaryon, while the ∆*arg-14::hph* mutant was a heterokaryon that required purification (presumably due to its arginine auxotrophy). The ∆*arg-14::hph* strain was streaked on FGS plates (2) containing 100 µg/mL arginine and 200 µg/mL hygromycin to facilitate the formation of colonies. Colonies were picked and inoculated onto VM slants containing 100 µg/mL arginine and 200µg/mL hygromycin and grown to generate cultures with conidia. This process was repeated to isolate homokaryotic ∆*arg-14::hph* mutant strains.

For DNA extraction, a small amount of *N. crassa* conidia or hyphal tissue was placed into 50 µL of extraction buffer composed of 100 mM TrisCl, 250 mM KCl, and 13 mM EDTA, pH 9.5. Samples were heated at 25˚C for 10 minutes and then at 95˚C for 10 minutes in a thermocycler to release genomic DNA. Samples were combined with 50 µL of a solution containing 3% BSA in sterile water. The Polymerase Chain Reaction (PCR) was performed using OneTaq® Quick-Load® 2x Master Mix with Standard Buffer (New England Biolabs M0486S) and primers (**Table S3**). Samples were subjected to agarose gel electrophoresis and PCR fragment size was determined by comparison to the Quick-Load® Purple 1 kb Ladder (New England Biolabs N0552S). To confirm the

status of the Δ*arg-14::hph* and Δ*vsb-1::hph* strains as both homokaryons and as Δ*arg-14::hph* or Δ*vsb-1::hph* mutants, two PCRs were performed to (1) assess the absence of the native gene locus and (2) to check for the presence of the hygromycin resistance gene (*hph*) at the locus (**Fig. S3B**).

RNA isolation and transcriptomics. For growth of cultures, either glass Erlenmeyer flasks treated with dichlorodimethylsilane (5% vol/vol in chloroform) or polycarbonate flasks were used to prevent hyphae from adhering to the inside walls of the flasks. Tissue was ground into a fine powder in liquid nitrogen using a mortar and pestle and total RNA extracted using TRIzol (Invitrogen, Waltham, MA) according to the manufacturer's recommendations. Samples containing 20 µg of RNA were treated with DNase I (New England Biolabs, Ipswich, MA) and then cleaned up using either the RNA Clean & Concentrator kit (Zymo Research, Irvine, CA) or the Monarch RNA Cleanup Kit (New England Biolabs) according to the manufacturer's recommendations. RNA quality was checked using agarose gel electrophoresis and by running a sample on the Agilent 2100 Bioanalyzer (Agilent, Santa Clara, CA). Three biological replicates were used for every strain.

RNA samples were sent to the University of California, Davis DNA Technologies and Expression Analysis Core Laboratory for Illumina poly-A mRNA library preparation and sequencing. Paired-end sequencing (2x150 bp) was performed using a NovaSeq 6000 with an S4 flow cell (Illumina, San Diego, CA). Raw reads were mapped against a generated index file via Kallisto version 0.46.1 (3) to generate raw counts and transcripts per million. Principal Component Analysis (PCA) was performed using R

(https://wwwR-project.org/) to check agreement between the biological replicates for each strain. Differential expression analysis was performed on raw counts using Deseq2 version 1.26.0 (4) and genes with at least a 2-fold change retained. Genes with p-values greater than 0.05 were not considered differentially expressed. Differential genes were then filtered to exclude genes with less than 10 transcripts per million in all four genotypes. FungiFun2 (https://sbi.hki-jena.de/fungifun/) was used to perform Functional Catalog (FunCat) analysis of the regulated genes in each genotype relative to wild type. Functional category data are available at https://elbe.hki-jena.de/fungifun/fungifun.php. Enzyme Commission (EC) numbers (5) were obtained from the Kyoto Encyclopedia of Genes and Genomes (KEGG) (6) or FungiDB (7, 8).

Metabolite extraction and liquid chromatography/mass spectrometry. Briefly, tissue collected as described above was lyophilized for 2 days at -80°C (Labconco, Kansas City, MO). Dried samples were pulverized at -80°C for one min with ceramic beads. A volume (1 mL) of extraction buffer (acetonitrile:methanol: water:isopropanol [3:3:2:2]) was added to 10 mg of weighed tissue and metabolites extracted (9). Liquid Chromatography/Mass Spectrometry analysis was performed using a TQ-XS triple quadrupole mass spectrometer (Waters, Milford, MA) coupled to an I-class UPLC system (Waters) as previously described (9).

Data analysis was performed using Skyline software (MacCoss Lab Software; https://www.skyline.ms/project/home/begin.view). There were ~210 potential molecules detected. Individual molecular peaks were manually checked for intensity, height above background, and retention time accuracy, and consistent peak boundaries were

assigned for each molecule between samples. Peaks were integrated using Skyline to analyze peak ratios. Experimental data was compared to preexisting standard LC-MS readings of metabolites to assign peaks to their corresponding small molecule. Four metabolites (proline, glutamine, phenylalanine and glycerophosphocholine) were present at levels higher than the threshold error and threshold concentrations, but were distinct spectra and therefore were detectable, but not quantifiable.

All mutants were compared to wild type, and relative abundance percentages were calculated by dividing the mutant value by the wild type value for each metabolite. Unpaired t-tests were performed using R Studio (https://wwwR-project.org/) to determine statistically significant differences between strains and to provide standard error for the five replicates for each strain. Significant differences are indicated according to p-values of $p<0.05$, $p<0.01$, and $p<0.001$. PCA was performed (https://wwwR-project.org/), using integrated peaks from the spectral analysis, with mean centering and scaling of data.

Measurement of cellular arginine and ornithine pools using column chromatography and colorimetric assays. For assessing levels of arginine and ornithine in hyphal cultures, liquid cultures were grown under the conditions described above and collected via vacuum filtration and frozen in liquid nitrogen before transferring to -80°C. Samples were lyophilized at -80°C for 2 days and then ground into a fine powder with a chilled mortar and pestle. For measurement of arginine and ornithine levels in macroconidia, strains were grown in VM agar flask cultures for 3 days at 30˚C in the dark and 5 days at 25˚C in the light. In order to avoid activation by water,

macroconidia were collected in oil as previously described (10), using Super Lube® H-3 Lightweight Oil ISO 68 (Synco Chemical Corporation Bohemia, NY). Approximately 40 mg of hyphal tissue or conidia was suspended in 1.2 mL of sterile water in a 1.5 ml tube and heated for 20 min in a 100°C water bath to extract metabolites. Samples were centrifuged at 21,000 x g for 5 min at 4°C and the supernatant was transferred to a new 1.5 mL tube and centrifuged again. A volume containing 1 mL of supernatant was collected and combined with 3 mL of 0.116 M citrate, pH 5.3. The entire volume was applied to a 2.3 mL DOWEX 50WX4-200 ion-exchange resin column (ThermoFisher Scientific, Waltham, MA). Fractions containing arginine and ornithine were collected as previously described (11). Ornithine and arginine levels were determined using colorimetric assays as previously described (12, 13) and normalized to the dry weight of the sample.

Enzymatic assays. Samples were lysed in a Bead-beater apparatus (Biospec Products, Bartlesville, OK) and centrifuged at 5000 x g and the supernatant retained (cell extract). Cell extracts were desalted by passaging over a Sephadex G25 (Sigma-Aldrich, St. Louis, MO) column at 4°C in an extraction buffer composed of 50 mM TrisCl, 1 mM PMSF, 1 mM DTT, and 1 mM EDTA. Protein concentrations of cell extracts were determined via the Bradford assay (Bio-Rad, Hercules, CA). All enzyme activities were obtained as nmoles of product/min/μg protein and then normalized to wild type.

The activity of N-acetylornithine-glutamate acetyl transferase was measured using a previously described method (14). The activity of N-acetylornithine deacetylase was determined in a reaction mix containing: 50 μM TrisCl, pH 7.5, 3 μM N-

acetylornithine, and 3 μM glutamate. The reaction was initiated by the addition of protein extract and proceeded for 10 minutes at 37˚C before quenching with the addition of Chinard assay reagent (25 mg ninhydrin, 0.4 mL of 6M phosphoric acid, and 1.6 mL glacial acetic acid) (13). Levels of ornithine were determined as previously described (13). The activity of ornithine transcarbamylase was assayed (15) in reaction mixtures containing 1.25 mg of extract protein, 6.15 mM carbamoyl phosphate, 6.15 mM ornithine, and 154 mM TrisCl, pH 9.0. The reaction was initiated by addition of protein extract and proceeded for 5 min at 25°C before quenching with 2 M perchloric acid (15). A previously described assay was used to measure the formation of the reaction product citrulline (16). Argininosuccinate synthetase activity was determined in reactions containing 2 mg of extract protein, 10 mM magnesium sulfate, 1 mM citrulline, 10 mM ATP, 20 mM L-aspartate and 50 mM TrisCl, pH 7.4. The reaction was initiated by addition of protein extract and proceeded for 15 min at 37°C before quenching with 1 M perchloric acid. Depletion of the citrulline substrate over time was determined using a previously described assay (16). The activity of argininosuccinate lyase was determined utilizing a previously described method (17). A volume of extract containing 1 mg of protein was added to a reaction containing 1 mM argininosuccinate, 0.2 mM manganese chloride and 20 mM sodium phosphate, pH 7.5. Reactions were incubated at 37°C for 10 min and quenched by boiling at 100°C for 10 min. Levels of the reaction product arginine were measured as described (12). Arginase activity was assayed as described (18, 19). Reactions containing 1 mg extract protein were preincubated in a 1 mL solution containing 1 mM manganese chloride and 20 mM glycine, pH 11.7, at 37°C for 10 min to allow arginase to bind the Mn^{2+} cofactor. The reaction was initiated by

addition of arginine to a final concentration of 25 mM and quenched after 30 min using 1 M perchloric acid. Levels of the reaction product urea were determined as previously described (20).

Detection of ARG-14 protein levels using western analysis. Tissue samples were flash frozen in liquid nitrogen, pulverized in a Qiagen Retsch TissueLyser (Hilden, Germany), and suspended in an extraction buffer composed of 50 mM TrisCl, 1 mM PMSF, 1 mM DTT, and 1 mM EDTA, pH 7.5. The lysate was centrifuged at 5000 x g and the supernatant retained (cell extract). Protein concentrations of cell extracts were determined via the Bradford assay (Bio-Rad, Hercules, CA). Samples containing 50 µg of protein were run on a 7.5% SDS-PAGE gel and Western blot analysis was performed as previously described (21). The upper portion of the membrane was stained using 1% amido black, 40% ethanol and 10% glacial acetic acid to verify equal loading. The ~70 kDa ARG-14 protein was detected using a rabbit polyclonal antibody (22).

SUPPLEMENTAL REFERENCES

1. **Colot HV, Park G, Turner GE, Ringelberg C, Crew CM, Litvinkova L, Weiss RL, Borkovich KA, Dunlap JC.** 2006. A high-throughput gene knockout procedure for *Neurospora* reveals functions for multiple transcription factors. Proc Natl Acad Sci U S A **103:**10352-10357.
2. **Davis RH, deSerres FJ.** 1970. Genetic and microbiological research techniques for *Neurospora crassa*. Methods Enzymol **71A:**79-143.
3. **Bray NL, Pimentel H, Melsted P, Pachter L.** 2016. Near-optimal probabilistic RNA-seq quantification. Nat Biotechnol **34:**525-527.
4. **Love MI, Huber W, Anders S.** 2014. Moderated estimation of fold change and dispersion for RNA-seq data with DESeq2. Genome Biol **15:**550.
5. **Biology NCotIUoBaM.** 1992. Enzyme Nomenclature 1992, 1st Edition. Academic Press, San Diego, CA.

6. **Kanehisa M, Furumichi M, Sato Y, Kawashima M, Ishiguro-Watanabe M.** 2023. KEGG for taxonomy-based analysis of pathways and genomes. Nucleic Acids Res **51:**D587-D592.
7. **Stajich JE, Harris T, Brunk BP, Brestelli J, Fischer S, Harb OS, Kissinger JC, Li W, Nayak V, Pinney DF, Stoeckert CJ, Jr., Roos DS.** 2012. FungiDB: an integrated functional genomics database for fungi. Nucleic Acids Res **40:**D675-681.
8. **Amos B, Aurrecoechea C, Barba M, Barreto A, Basenko EY, Bazant W, Belnap R, Blevins AS, Bohme U, Brestelli J, Brunk BP, Caddick M, Callan D, Campbell L, Christensen MB, Christophides GK, Crouch K, Davis K, DeBarry J, Doherty R, Duan Y, Dunn M, Falke D, Fisher S, Flicek P, Fox B, Gajria B, Giraldo-Calderon GI, Harb OS, Harper E, Hertz-Fowler C, Hickman MJ, Howington C, Hu S, Humphrey J, Iodice J, Jones A, Judkins J, Kelly SA, Kissinger JC, Kwon DK, Lamoureux K, Lawson D, Li W, Lies K, Lodha D, Long J, MacCallum RM, Maslen G, McDowell MA, et al.** 2022. VEuPathDB: the eukaryotic pathogen, vector and host bioinformatics resource center. Nucleic Acids Res **50:**D898-D911.
9. **Carrillo AJ, Halilovic L, Hur M, Kirkwood JS, Borkovich KA.** 2022. Targeted Metabolomics Using LC-MS in Neurospora crassa. Curr Protoc **2:**e454.
10. **Kim JD, Kaiser K, Larive CK, Borkovich KA.** 2011. Use of 1H nuclear magnetic resonance to measure intracellular metabolite levels during growth and asexual sporulation in *Neurospora crassa*. Eukaryot Cell **10:**820-831.
11. **Basabe JR, Lee CA, Weiss RL.** 1979. Enzyme assays using permeabilized cells of Neurospora. Anal Biochem **92:**356-360.
12. **Jorpes E, Thoren S.** 1932. The use of the Sakaguchi reaction for the quantitative determination of arginine. Biochem J **26:**1504-1506.
13. **Chinard FP.** 1952. Photometric estimation of proline and ornithine. J Biol Chem **199:**91-95.
14. **Staub M, Denes G.** 1966. Mechanism of arginine biosynthesis in Chlamydomonas reinhardti. I. Purification and properties of ornithine acetyltransferase. Biochim Biophys Acta **128:**82-91.
15. **Davis RH.** 1962. A mutant form of ornithine transcarbamylase found in a strain of Neurospora carrying a pyrimidine-proline suppressor gene. Arch Biochem Biophys **97:**185-191.
16. **Koritz SB, Cohen PP.** 1954. Colorimetric determination of carbamylamino acids and related compounds. J Biol Chem **209:**145-150.
17. **Farrell K, Overton S.** 1987. Characterization of argininosuccinate lyase (EC 4.3.2.1) from Chlamydomonas reinhardtii. Biochem J **242:**261-266.
18. **Borkovich KA, Weiss RL.** 1987. Purification and characterization of arginase from *Neurospora crassa*. J Biol Chem **262:**7081-7086.
19. **Davis RH, Mora J.** 1968. Mutants of Neurospora crassa deficient in ornithine-delta-transmainase. J Bacteriol **96:**383-388.
20. **Archibald RM.** 1945. Chemical characteristics and physiological roles of glutamine. Chem Rev **37:**161-208.
21. **Krystofova S, Borkovich KA.** 2005. The heterotrimeric G-protein subunits GNG-1 and GNB-1 form a Gbetagamma dimer required for normal female

fertility, asexual development, and galpha protein levels in *Neurospora crassa*. Eukaryot Cell **4:**365-378.

22. **Yu YG, Turner GE, Weiss RL.** 1996. Acetylglutamate synthase from Neurospora crassa: structure and regulation of expression. Mol Microbiol **22:**545-554.

SUPPLEMENTAL TABLES

Table S1. Metabolites with different relative abundances in the mutants vs. wild type

		Lower abundance			Higher abundance		
**Class**	**Total Detected**	**Δ*gna-1***	**Δ*gna-3***	**Δ*ric8***	**Δ*gna-1***	**Δ*gna-3***	**Δ*ric8***
Acetyl amino acid	4			3	2	1	
Amino acid	29	5	1	16	1	9	3
Electron Carrier	4	2	1	4			
Organic acid	7	1	1	3	1	2	1
Oxidative stress	5			2			1
Purine	21	4		7	1	3	5
Pyrimidine	8			2		1	
TCA cycle	4			1	1		2
Urea cycle	4	1		2			1
Misc.	35	3	4	6	3	6	9
**Total**	**121**	**16**	**7**	**46**	**9**	**22**	**22**

Table S2. Pools of arginine and ornithine in macroconidia compared to wild type hyphae.

Metabolite	Strain	% Wild-type hyphae	*SE	p-value (compared to wild type hyphae)
Arginine	Wild type	0.33	0.016	2.0×10^{-14}
	Δ*gna-1*	0.26	0.016	2.0×10^{-14}
	Δ*gna-3*	8.1	0.26	1.1×10^{-13}
	Δ*ric8*	8.6	1.2	1.3×10^{-13}
Ornithine	Wild type	Not Detectable	-	-
	Δ*gna-1*	Not Detectable	-	-
	Δ*gna-3*	Not Detectable	-	-
	Δ*ric8*	Not Detectable	-	-

*SE-Standard error

Table S3. Primers used for PCR verification of the Δ*vsb-1* and Δ*arg-14* mutations.

Primer Name	Sequence
vsb-1 RV	5’ GCCTGGACGCGCATTCTGCT 3’
hph internal FW	5’ TCTTAGCCAGACGAGCGGGTTCGGC 3’
arg-14 upstream FWD	5’ CGAGGGCCACGTCCTAGCTGAGAAG 3’
arg-14 internal RV	5’ CGCACAAACATGGCTCATGAATCTG 3’
hph internal RV	5’ CGCCCCAGCACTCGTCCGAGG 3’

SUPPLEMENTAL FIGURES

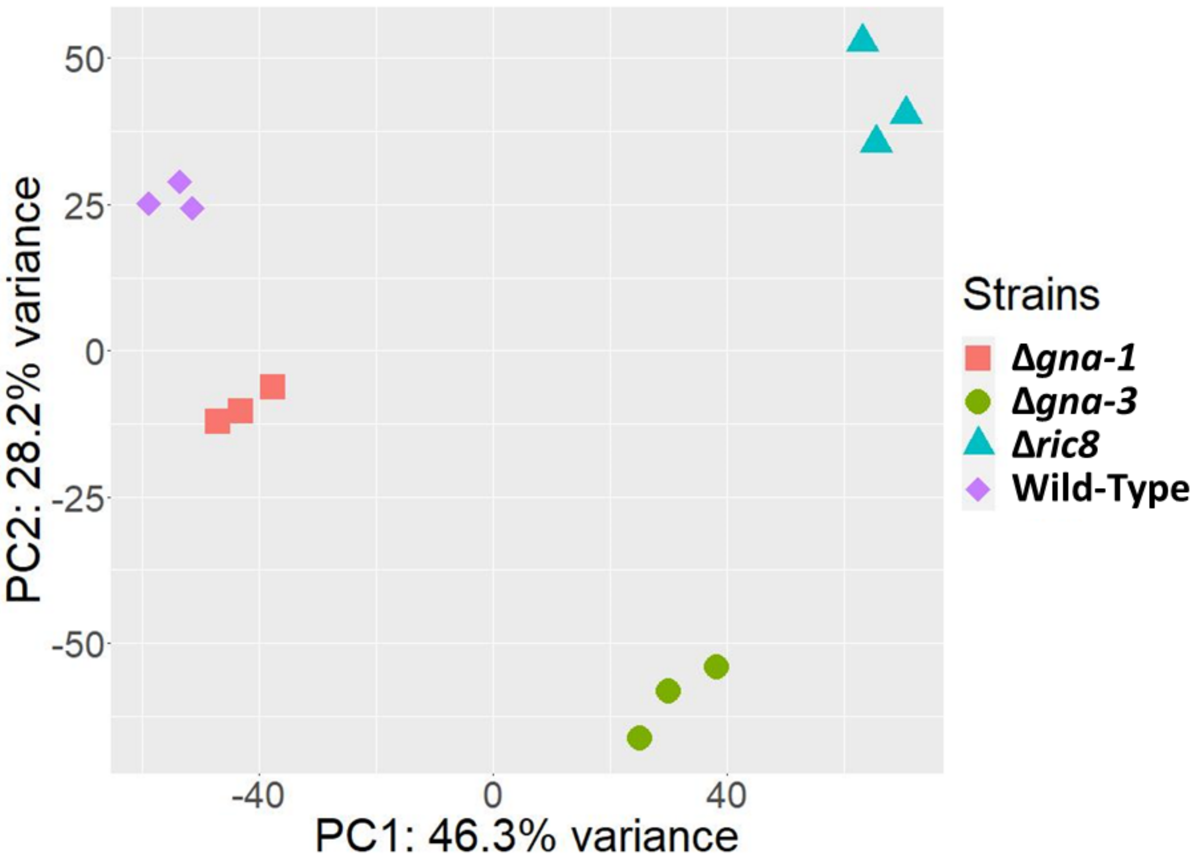

Figure S1. Principal component analysis of RNAseq data.

A 2-dimensional Principal Component Analysis (PCA) was performed in R (https://wwwR-project.org/) utilizing data from the Kallisto 0.46.1 alignment of RNAseq reads to the reference genome OR74A. Data included reads for three biological replicates for each of the four strains.

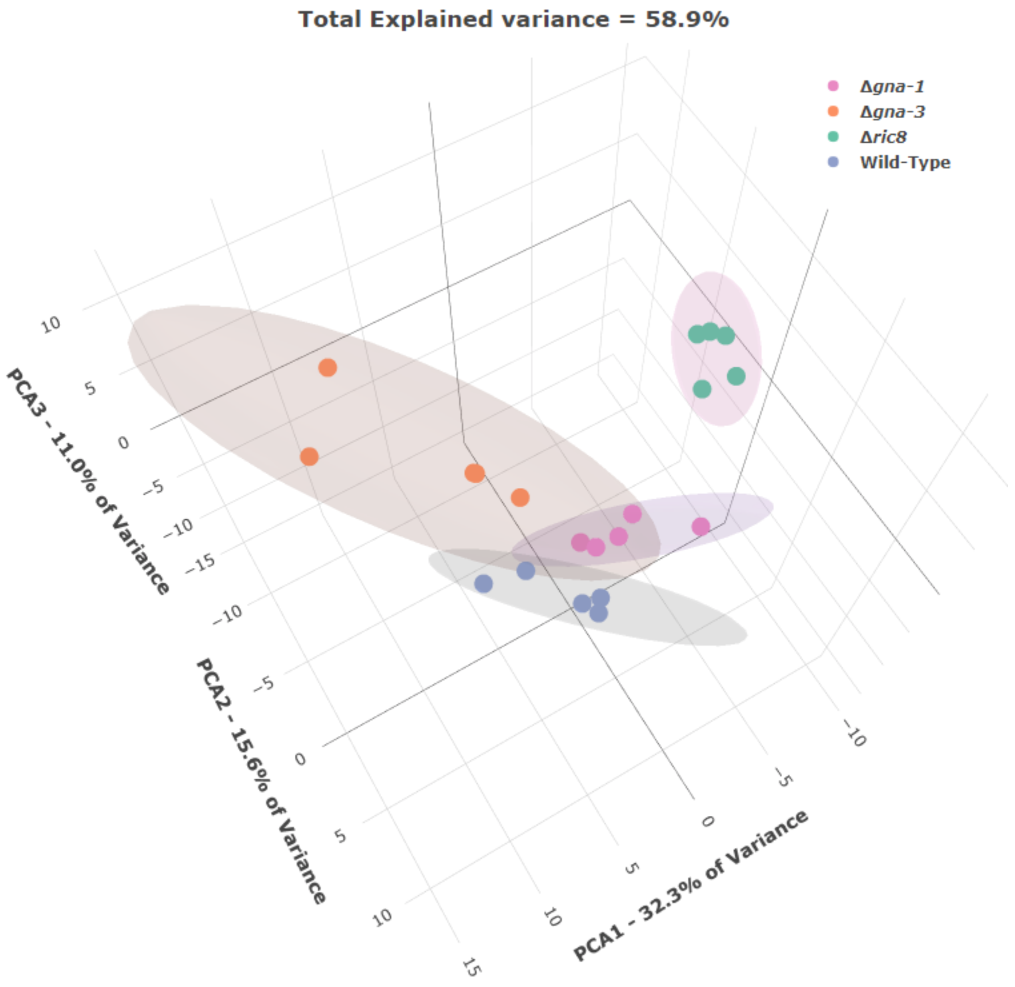

Figure S2: Principal component analysis of metabolite relative abundances.

Utilizing relative abundances from the LC-MS spectra analysis, a 3-dimensional Principal Component Analysis (PCA) was performed using R (https://www.R-project.org/). A total of five biological replicates were used for each of the four strains.

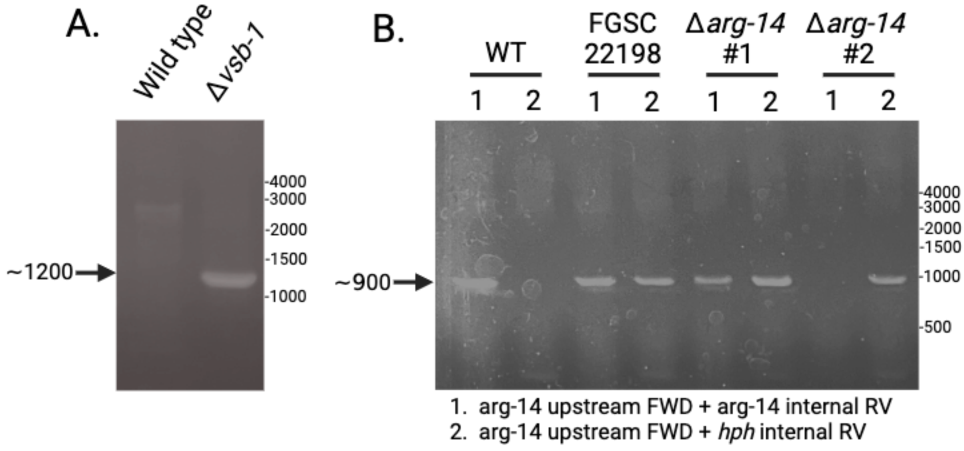

Figure S3. PCR verification of the ∆*vsb-1:hph* and ∆*arg-14:hph* knockout mutants.

Genomic DNA was extracted from wild type, ∆*vsb-1* and ∆*arg-14* strains and PCR was performed using the primers described in Table S2 as described in the Supplemental Methods.

A. **∆*vsb-1::hph* PCR verification.** A 1276 bp PCR product was expected for the ∆*vsb-1* mutation. A band of ~1200bp was observed in the ∆*vsb-1* strain, but absent in reactions using DNA from the wild type strain, validating the genotype of the ∆*vsb-1* mutant.
B. **∆*arg-14::hph* PCR verification.** Confirmation that an ∆*arg-14* homokaryotic mutant had been isolated was verified using two different PCRs. (1) The first PCR had an expected product size of 865 bp and tested for the presence of the wild type (WT) *arg-14* locus. A band of ~900 bp is present in the wild type and the ∆*arg-14::hph* mutant heterokaryon (FGSC22198). This band, however, is absent in the purified strain (∆*arg-14* #2), indicating that this strain does not have the *arg-14* gene at the native locus. (2) A second PCR was performed to confirm that the hygromycin B resistance gene (*hph*) was present at the *arg-14* locus. A PCR product of 898 bp was expected if the *hph* gene was present in place of the native *arg-14* locus. A band of ~900 bp was observed in both the ∆*arg-14::hph* heterokaryon and the ∆*arg-14::hph* homokaryotic strain that was not present in the wild-type strain, confirming the knockout.

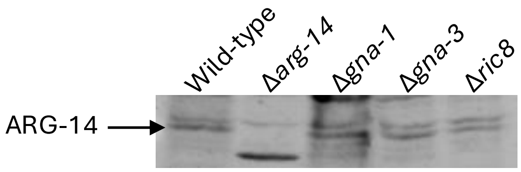

Figure S4: Levels of ARG-14 protein in Wild-type, ∆*gna-1,* ∆*gna-3,* and ∆*ric8* mutants. Samples containing 50 µg of cell extract protein were subjected to Western analysis using an ARG-14 antibody as described in the **Supplemental Methods**. The arrow marks the position of the ~70 kDa ARG-14 protein.
